# Sustained function and quality of life 20 years after LCS mobile-bearing total knee arthroplasty: a retrospective cohort study

**DOI:** 10.1007/s00402-025-05983-w

**Published:** 2025-07-09

**Authors:** Amir Koutp, Sophie Plakolb, Lukas Leitner, Rene Schroedter, Andreas Leithner, Patrick Sadoghi

**Affiliations:** 1 Department of Orthopaedics and Trauma, Medical University of Graz, Graz, Austria; 2 Department of Orthopaedics and Trauma Surgery, Musculoskeletal University Center Munich (MUM), LMU University Hospital, Munich, Germany

**Keywords:** Total knee arthroplasty, Mobile bearing, Long-term outcome

## Abstract

**Purpose:**

The aim of this study was to assess the long-term clinical outcome, quality of life (QoL), and complications associated with low-contact-stress (LCS) mobile-bearing total knee arthroplasty (TKA) with a minimum follow-up of 20 years.

**Methods:**

This is a retrospective cohort study based on a previous report that initially included 108 patients with 138 prostheses. For a minimum follow-up of 20 years, reevaluation was conducted on the remaining cohort of 15 patients, as 80 had deceased, 11 were lost to follow-up and 2 were excluded due to revision surgery. Patients were assessed for quality of life (QoL), clinical outcomes, and complications. Patient reported outcome measures were obtained via questionnaires, and range of motion (ROM) was clinically evaluated. Descriptive and explorative data analysis was performed.

**Results:**

During the follow-up period, there were fifteen revision surgeries, however the prostheses had an overall survival of 22 years. Twenty years postoperatively, active range of motion (ROM) remained comparable to 10 years, with no significant differences between females (98.46° ± 27.72° vs. 96° ± 16.7°; *p* = 0.105) or males (90° ± 14.14° vs. 95° ± 17.3°). WOMAC scores at 20 years (83.77 ± 11.01) were comparable to 10 years (81.46 ± 17.88). Knee Society Score (KSS) pain scores showed no significant changes either (females: 69 ± 20.9, males: 76 ± 24.4) and 20 years (73.81 ± 25.86; *p* = 0.398). Function scores declined significantly over time, from 80.38 ± 19.20 to 51.54 ± 31.65 in females and from 79 ± 24 to 37.5 ± 53.03 in males (*p < 0.05*), which is in line with the patients age and natural decline.

**Conclusion:**

The findings of this 20-year follow-up suggest that the LCS TKA can provide durable outcomes and sustained quality of life benefits in surviving patients. The observed decline in function may reflect age-related changes rather than implant performance.

## Introduction

The low-contact-stress (LCS) mobile-bearing total knee prosthesis (Johnson & Johnson, New Brunswick, NJ, USA; previously DePuy, Warsaw, IN, USA) was long regarded as a gold standard in total knee arthroplasty (TKA) due to its design, which aimed to provide excellent long-term functional results for both female and male patients [1–5]. One of the theoretical advantages of the LCS system lies in its focus on achieving neutral mechanical alignment, where the mechanical axis passes through the mid-line of the knee. This alignment is designed to ensure even load distribution across the medial and lateral femorotibial compartments, thereby reducing the risk of implant loosening and wear over time [6–10]. Despite the emergence of various alignment philosophies and alternative prosthetic designs, the LCS prosthesis continues to be implanted in some countries and remains a relevant topic of study, particularly given its widespread use in earlier decades [2, 11].

Several studies have reported long-term follow-up data of over 10 years for the LCS prosthesis, and some have documented survival rates exceeding 20 years [2, 12, 13]. However, there is a notable scarcity of research focusing on functional outcomes and quality of life associated with this prosthesis at long-term follow-up. While prosthesis longevity remains an important metric, it is equally critical to assess the quality of life of patients, especially as TKA is increasingly performed in younger patients [14]. For these individuals, long-term functional performance and daily living improvements are paramount, underscoring the need for a broader perspective in evaluating TKA success.

Therefore, the aim of this study was to assess the clinical outcomes, quality of life, and complications associated with LCS mobile-bearing TKA with a minimum follow-up of 20 years.

Our hypothesis was that the LCS mobile-bearing TKA provides satisfactory outcomes in terms of clinical performance, quality of life, and complication rates, even with a minimum follow-up of 20 years.

## Patients and methods

### Study design and recruitment

This is a retrospective Level III cohort study. Utilizing the clinic database and the data from a previously published study of our group [1], the same patient cohort (108 patients with 138 prostheses) was reevaluated after further 10 years, following a previously published 10 year follow-up.

Due to the advanced age of participants at the time of evaluation, 80 patients were deceased. Eleven patients were lost to follow-up, and two patients had undergone revision surgery due to aseptic loosening. Fifteen TKAs were in situ at final follow up, including 13 females and 2 males. The study was approved by the institutional ethics committee (23–284 ex 10/11). All patients provided written informed consent for the use of their anonymized data in research, in accordance with the Declaration of Helsinki.

### Surgical technique and rehabilitation

All prostheses were implanted in a single institution under general or epidural anaesthesia by three different orthopaedic surgeons, as previously described [15, 16]. Postoperatively, patients were allowed full weight-bearing. Continuous passive motion was initiated two days after surgery, and all patients were discharged within 10 to 14 days. Patients were referred to outpatient rehabilitation programs until the sixth postoperative week.

### Pain management

Pain management included intravenous administration of 75 mg diclofenac and 30 mg orphenadrine, as well as oral pantoprazole (40 mg) or intravenous metamizole (1 g). Pantoprazole was continued orally, and intramuscular piritramide (7.5 mg) was provided as an additional treatment option.

### Radiographic evaluation

Standardized anteroposterior (AP) and lateral radiographs of the knee were obtained at the final follow-up to assess for radiolucent lines. Evaluation was performed using the zonal system described for tibial and femoral components, with Zones 1–7 assessed on AP tibial view, Zone 1–3 on lateral tibial views, and Zones 1–7 on the lateral femoral view. Radiolucency was defined as any non-progressive line visible at the bone–implant interface and was recorded as present or absent [17]. The presence of radiolucent lines was interpreted by two independent observers. Radiographic findings were descriptively summarized and not used to guide treatment decisions unless progressive changes were evident.

### Patient reported outcome measurements (PROMS)

At a minimum follow-up of 20 years, patients were clinically assessed for quality of life (QoL), clinical outcomes, and complications. The Western Ontario and McMaster Universities Osteoarthritis Index (WOMAC) and Knee Society Score (KSS) were obtained via questionnaires. Range of motion (ROM) was clinically evaluated.

### Data analyses

Descriptive and exploratory data analyses were performed. The Mann-Whitney U test was used to compare demographic parameters between groups, as the data were not normally distributed. Main endpoints had been evaluated in a previous report of the same cohort according to sample size and power. All statistical analyses were conducted using SPSS 29 (IBM Corp., Armonk, NY, USA), and p-values < 0.05 were considered statistically significant. Given the reduced sample size at long-term follow-up, the study was not powered to detect small to moderate differences in PROMs beyond 20 years. Therefore, nonsignificant findings should be interpreted with caution.

In addition to Kaplan-Meier analysis for prosthesis survival, we performed a competing risks analysis to differentiate between prosthesis revision and patient mortality. Complications that led to surgical revision, including aseptic loosening, infection, wear, instability, and fracture, were considered revision events. In the Kaplan-Meier analysis, patients who were lost to follow-up were censored at their last documented point of contact. All cumulative incidence curves and survival analyses were performed using R version 4.3.1 (R Foundation for Statistical Computing, Vienna, Austria) with the survival and cmprsk packages.

## Results

### Demographic data

At the time of follow-up (20 to 24 years), the average age of female participants was 79 years (range: 60–92 years), while male participants averaged 74 years (range: 61–86 years).

One female and one male patient had undergone revision surgery due to aseptic loosening at a follow-up of 22 years respectively. Eighty patients deceased in the period from 10 to 20 years of follow-up without relation to the index procedure and eleven patients were lost to follow-up.

**Fig. 1:**
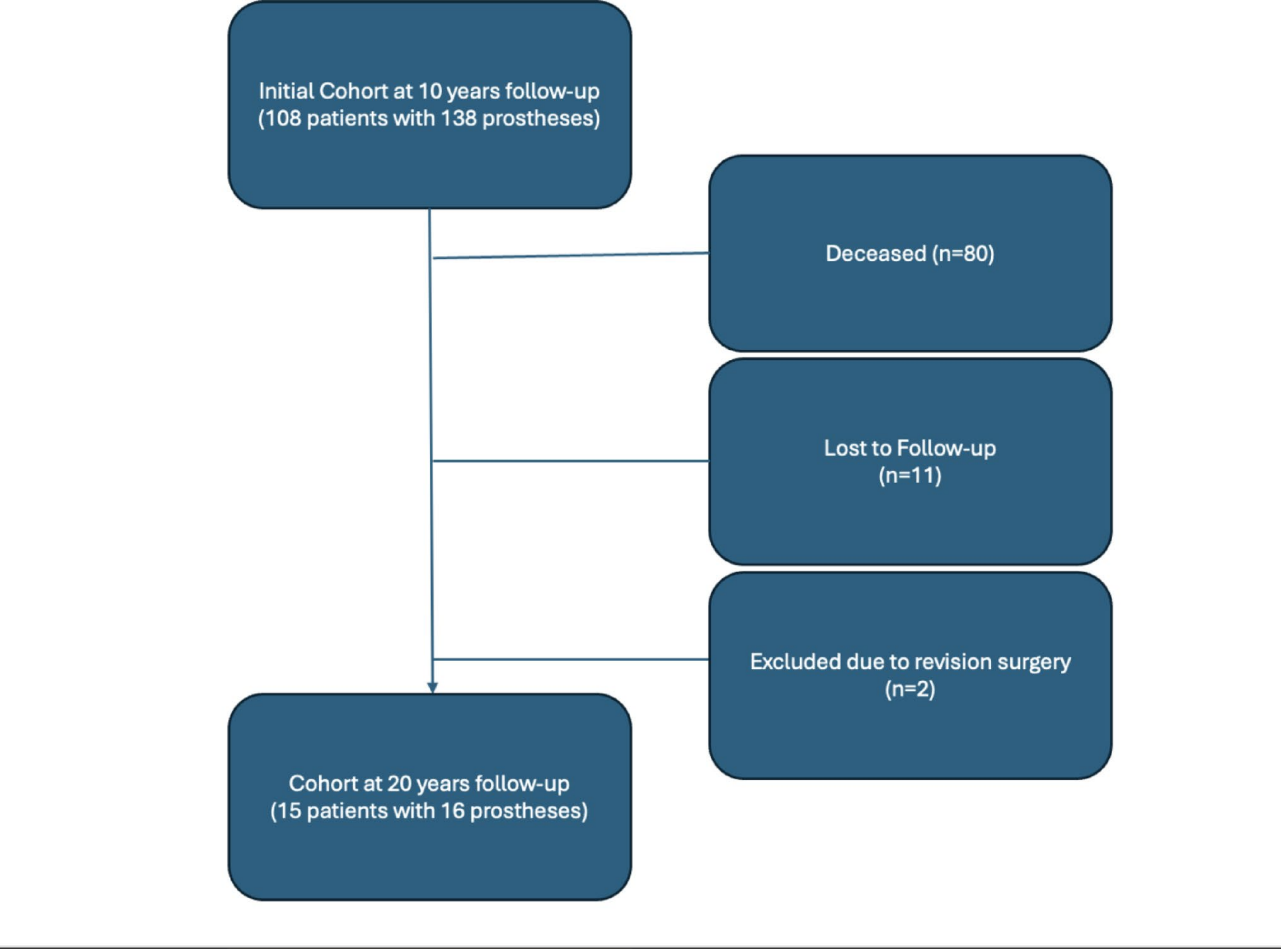
CONSORT diagram illustrating the patient selection and follow-up process. The initial cohort consisted of 108 patients with 138 prostheses at the 10-year follow-up. Of these, 80 patients deceased, 11 were lost to follow -up, and 2 patients were excluded following revision surgery. The final cohort at the 20-year follow-up included 15 patients with 16 prostheses

The patient selection process and follow-up details are illustrated in Fig. 1, which provides an overview of the exclusions, and the final cohort included in the 20-year follow-up analysis.

While patients who underwent revision were excluded from PROMs analysis, they were included in the survival and competing risk analysis to reflect the occurrence and timing of mechanical complications.

### Radiographic findings

Radiographic analysis was available for all 15 patients at final follow-up. Radiolucent lines were identified in at least one tibial zone in 11 out of 16 knees (68.8%), most frequently in the medial tibial zones (Zones 1–4). Femoral radiolucencies were rare, occurring in only one case in Zones 1 and 2. No progressive radiolucent lines or signs of loosening were observed. None of the patients with radiolucent lines reported pain or functional decline attributable to these findings, and no revisions were indicated based on radiographic assessment alone. These results suggest that while radiolucent lines were relatively common, they were clinically insignificant in the long term.

### Clinical outcomes

#### Range of motion (ROM)

At 10 years, the average passive range of motion was 99° ± 20.2° for female patients (*n* = 82) and 101° ± 17.9° for male patients (*n* = 26), with no statistically significant difference (*p = 0.962*). Similarly, active range of motion showed no significant difference, with females achieving 96° ± 16.7° and males 95° ± 17.3° (*p = 0.860*) at 10 years follow-up. At 20 years the active range of motion of males was 90°± 14.14° and 98.46° ± 27.72 for females without significant difference (*p = 0.105*).

*WOMAC Scores*.

**Fig. 2:**
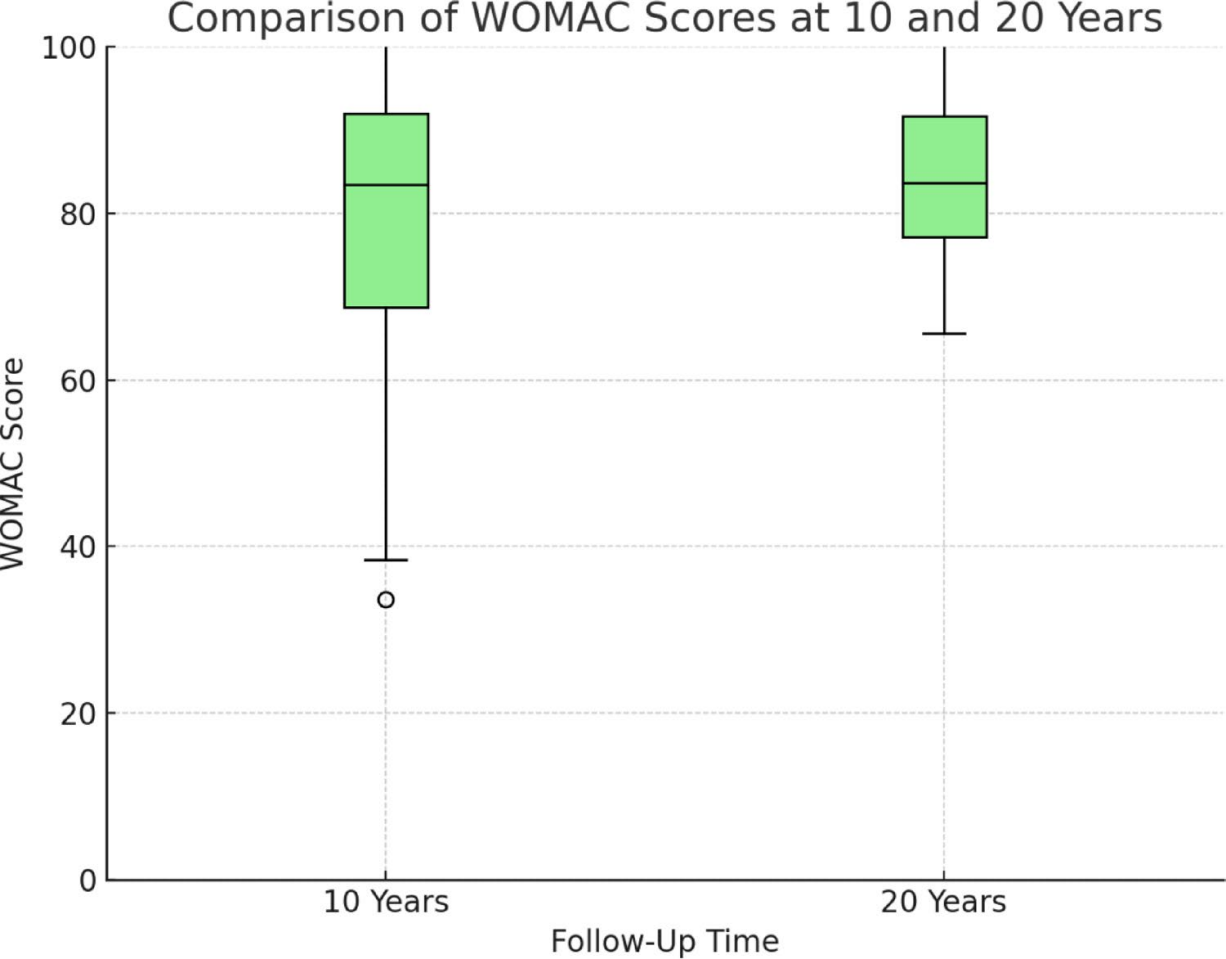
WOMAC Scores

For females, the WOMAC score at 20 years (83.77 ± 11.01) was slightly higher compared to 10 years (81.46 ± 17.88), indicating a marginal decline in outcome, though the difference was not statistically significant (*p = 0.305*) as depicted in Fig. 2. Although the change in WOMAC scores was not statistically significant, the magnitude of difference also falls below the minimally clinically important difference (MCID), which is typically considered to be 10–12 points for the WOMAC index.

Boxplot Comparison of WOMAC Scores at 10-Year and 20-Year Follow-Ups.

This figure displays the WOMAC scores for patients at the 10-year and 20-year follow-ups. The median scores are represented by the horizontal line inside each box, while the interquartile range (IQR) is shown by the box. Whiskers extend to the minimum and maximum values, excluding outliers (denoted by asterisks). A higher WOMAC score indicates worse pain and function. There is no statistically significant difference between the two time points (*p* = 0.305).

#### Knee Society Score (KSS)

At 10 years, the KSS pain score was slightly higher for male patients (76 ± 24.4) compared to females (69 ± 20.9), though the difference was not statistically significant *(p = 0.442)*. At 20 years, the KSS pain score was 73.81 ± 25.86, and the difference compared to the 10-year follow-up was not statistically significant *(p = 0.398)*.

**Fig. 3:**
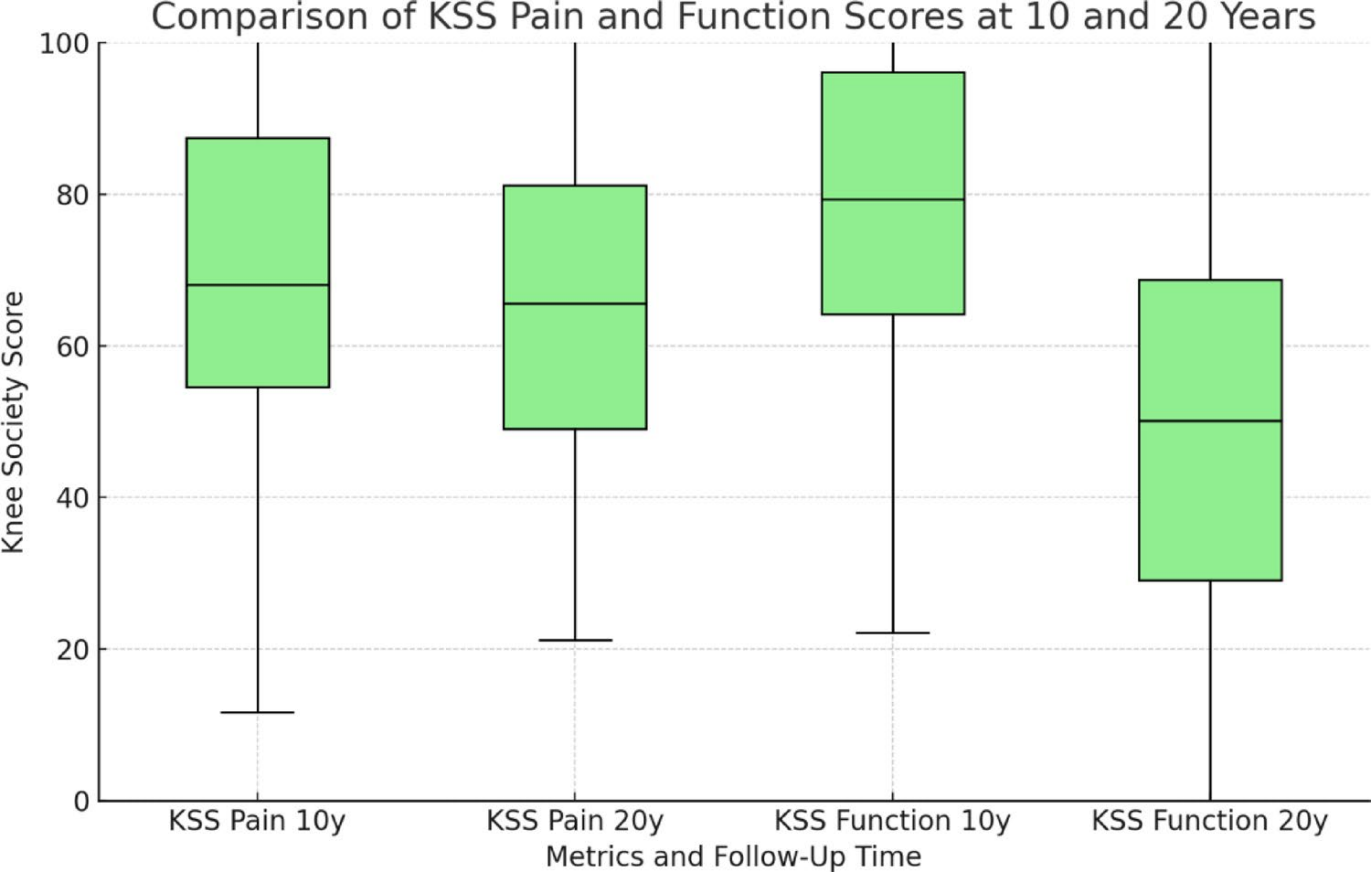
KSS Pain and Function Scores

Function Scores: In comparison to the results at 10 years female patients’ function scores decreased significantly from 80.38 ± 19.20 at 10 years to 51.54 ± 31.65 at 20 years and for males from 79 ± 24 at 10 years to 37.5 ± 53.03 at 20 years *(p < 0.05)* as shown in Fig. 3.

Boxplot Comparison of KSS Pain and Function Scores at 10-Year and 20-Year Follow-Ups.

This figure compares the KSS Pain and Function scores at the 10-year and 20-year follow-ups. Separate boxplots for each time point (10 years and 20 years) and measure (Pain and Function) are shown. The median values are indicated by the line within each box, and the IQR is shown by the box boundaries. Whiskers represent the range of data excluding outliers (denoted by asterisks). While KSS Pain scores did not show significant differences between time points (*p* = 0.398), KSS Function scores significantly decreased from 10 to 20 years (*p* < 0.05).

Table 1 provides a detailed summary of patient-reported outcome measures (PROMs) and range of motion (ROM).

**Table 1:** Comparison of PROMS at 10 and 20 years follow up

PROMS	10-Year Follow-Up (Mean ± SD)	20-Year Follow-Up (Mean ± SD)	*p*-Value
WOMAC	81.46 ± 17.88	83.77 ± 11.01	0.305
KSS Pain	72.50 ± 24.40	73.81 ± 25.86	0.398
KSS Function	79.50 ± 21.60	51.54 ± 31.65	< 0.05
Active ROM (°)	95 ± 17.3	90 ± 14.14	0.105

**Fig. 4:**
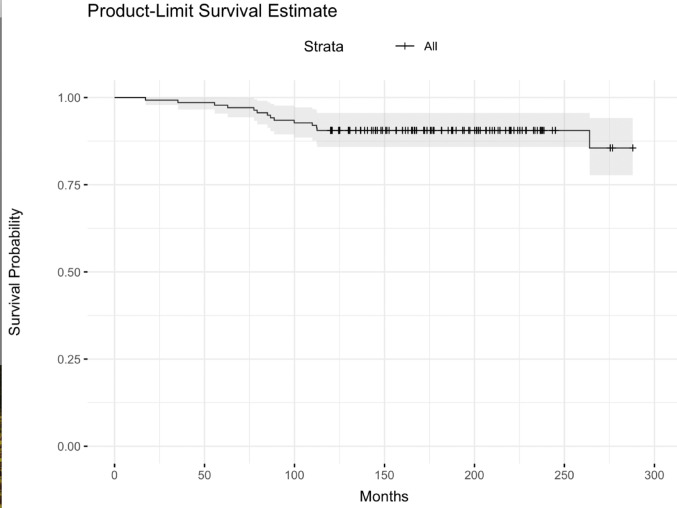
Kaplan-Meier survival curve showing prosthesis survival over a 25-year period. The endpoint was revision surgery. Patients who died, were lost to follow-up, or remained event-free were censored. The survival curve demonstrates excellent long-term prosthesis durability, with only two revisions observed during follow-up (survival probability: 98.15% at 24 years)

Kaplan-Meier analysis demonstrated excellent prosthesis survival over 25 years. A total of 15 revisions were recorded, including 13 complications between years 1 and 10 based on retrospective analysis of clinical records and consistent with our previously published data, and two cases of aseptic loosening at 22 years. These events resulted in a prosthesis survival probability of 89.13% at final follow-up (Fig. 4).

**Fig. 5:**
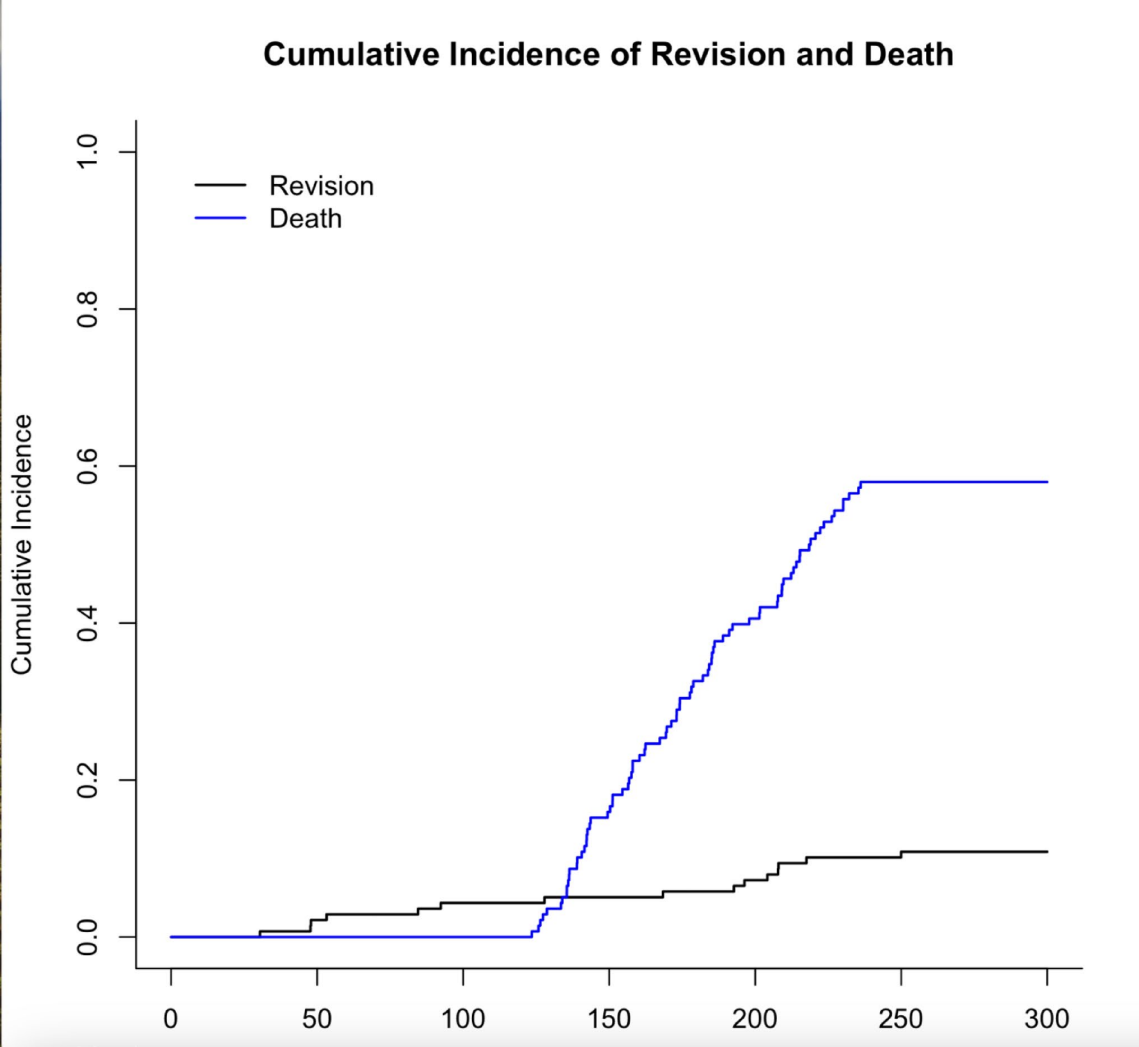
Cumulative incidence of revision and death over 25 years. The competing risks model illustrates a cumulative revision risk of 9.4% in 138 TKAs, including five cases of aseptic loosening, three late infections, three inlay wear cases, one instability, and one periprosthetic fracture. Two patients underwent revision for aseptic loosening at 22 years. Death was treated as a competing event and increased steadily between years 10 and 20

To account for the influence of competing events, a cumulative incidence function (CIF) analysis was performed. As shown in Fig. 5, the cumulative incidence of revision rose steadily during the first 10 years, reflecting 13 early complications that required surgical intervention. A smaller rise was observed at 22 years due to two additional revisions for aseptic loosening. The cumulative incidence of revision reached approximately 10.9% at 25 years. These findings suggest that while mechanical failure was rare, mortality significantly influenced long-term revision-free survival estimates.

These findings support the interpretation that prosthetic complications were rare and that age-related mortality was the predominant factor influencing long-term outcomes.

## Discussion

The current analysis extends a previously published 10-year follow-up and provides rare insights into long-term performance beyond two decades. Despite the small remaining cohort, the continuity of follow-up strengthens the clinical relevance of the findings. Our findings demonstrate that the LCS TKA provides durable and effective results over two decades. Despite the observed decline in certain functional parameters, such as the KSS function score, these changes are largely attributable to age-related factors rather than prosthesis-related issues. We present one of the first studies including PROMS and QoL at this follow-up period. While the absolute number of events is low in the later years due to natural attrition, this reflects a realistic long-term trajectory in elderly TKA populations and underscores the importance of accounting for mortality in outcome studies.

At 20 years post-implantation, patients maintained high scores for quality of life (QoL) as measured by the WOMAC and KSS pain scales, which were comparable to the 10-year follow-up results. The decline observed in the KSS function score is likely reflective of the natural aging process and associated mobility limitations. Many participants required walking aids or had limited walking capacity, factors that are integrated into the KSS function score. Importantly, these limitations were not directly attributable to prosthetic failure but rather to the broader context of patient aging and comorbidities.

The complication rate at 20 years was low, with two cases of aseptic loosening managed successfully through revision surgeries. These results reaffirm the durability of the LCS TKA design, which continues to provide satisfactory outcomes decades after implantation, as demonstrated by several other studies [2, 5, 6, 12, 13, 18–21]. The absence of major quality of life or satisfaction declines supports the long-term reliability of this prosthesis.

Additionally, radiographic evaluation revealed non-progressive radiolucent lines in a subset of cases, most commonly around the medial tibial zones, without clinical correlation or evidence of loosening. These findings further support the structural integrity and long-term fixation of the implant, even two decades post-implantation.

The observed functional decline among participants highlights the impact of aging on long-term outcomes. As patients enter their 70s and 80s, age-related factors such as reduced strength, increased reliance on assistive devices, and general frailty inevitably influence performance metrics. This is further confirmed by Kakiage et al. and numerous other studies, which emphasize the importance of interpreting long-term prosthetic outcomes in the context of patient aging rather than attributing them solely to prosthetic design [22–28].

The Kaplan-Meier survival curve and competing risk analysis based on observed patient mortality closely matched the trends reported in this study. A mean prosthesis survival time of 22 years was confirmed, consistent with previous long-term findings. Importantly, the competing risk model highlights that the majority of patients were lost to follow-up due to age-related mortality rather than prosthetic complications.

This supports our conclusion that the observed decline in function is predominantly due to aging and associated limitations, not prosthesis failure. Long-term outcome studies in elderly populations must account for competing mortality to avoid overestimating implant performance.

This study adds to the limited body of evidence on 20-year follow-up outcomes for LCS TKA including QoL and PROMS. While studies on gender-specific prostheses have highlighted anatomical differences between male and female knees, our findings indicate that a standard prosthesis can provide durable and satisfactory results for female patients over two decades [1].

Future research should prioritize recruiting larger and more balanced cohorts to allow for reliable gender-specific comparisons. Additionally, studies should investigate other factors that influence long-term outcomes, including patient weight, activity levels, and comorbid conditions. The integration of advanced imaging and gait analysis may also provide further insights into the factors contributing to functional outcomes after knee arthroplasty.

### Limitations

The primary limitation of this study is the small sample size of 15 participants, which increases the likelihood of random effects and limits the generalizability of the findings. However, this is due to the natural aging of the selected patient population. Furthermore, the uneven gender distribution, with only two male participants compared to 13 females, restricts the study’s capacity to draw meaningful conclusions about gender-specific differences in clinical outcomes, which was an aim of our previous report. Another limitation is the advanced age of the participants, as many were affected by age-related conditions that impacted their mobility and function, independent of their knee prosthesis. These factors directly influenced the collected scores (e.g., WOMAC, KSS) and reduced their interpretability as measures of prosthesis performance. Comparisons to a younger, healthy control group or the use of age-adjusted corrections in future studies could enhance the validity of these findings. Additionally, the generalizability of our findings is limited by the considerable loss to follow-up due to patient mortality over the 20-year period. This attrition, while expected in an elderly cohort, reduces the statistical power and impacts the extrapolation of results to younger or more diverse populations. Cases lost to follow-up were censored at the time of last contact in the survival analysis to minimize bias and maintain methodological robustness. Despite these limitations, this study provides valuable insights into the long-term performance of the LCS TKA, with a minimum follow-up of 20 years, offering a rare perspective on the durability and efficacy of this prosthesis over extended periods with a focus on PROMs and QoL, which has not been published sufficiently before.

## Conclusion

The findings of this 20-year follow-up suggest that the LCS TKA can provide durable outcomes and sustained quality of life benefits in surviving patients. The observed decline in function may reflect age-related changes rather than implant performance.

## Data Availability

No datasets were generated or analysed during the current study.

## References

[1] Kastner N, Aigner BA, Meikl T, Friesenbichler J, Wolf M, Glehr M et al (2014) Gender-specific outcome after implantation of low-contact-stress mobile-bearing total knee arthroplasty with a minimum follow-up of ten years. Int Orthop 38:2489–2493. 10.1007/s00264-014-2453-4 25027979 10.1007/s00264-014-2453-4

[2] Buechel FF, Buechel FF, Pappas MJ, D’Alessio J Twenty-year evaluation of meniscal bearing and rotating platform knee replacements. Clin Orthop Relat Res 2001:41–50. 10.1097/00003086-200107000-00008 10.1097/00003086-200107000-00008 11451131

[3] Sadoghi P, Leithner A, Weber P, Friesenbichler J, Gruber G, Kastner N et al (2011) Radiolucent lines in low-contact-stress mobile-bearing total knee arthroplasty: a blinded and matched case control study. BMC Musculoskelet Disord 12:142. 10.1186/1471-2474-12-142 21714916 10.1186/1471-2474-12-142 PMC3152942

[4] Stiehl JB (2002) World experience with low contact stress mobile-bearing total knee arthroplasty: a literature review. Orthopedics 25. 10.3928/0147-7447-20020202-04 10.3928/0147-7447-20020202-04 11866156

[5] Callaghan JJ, Squire MW, Goetz DD, Sullivan PM, Johnston RC (2000) Cemented rotating-platform total knee replacement a nine to twelve-year follow-up study*. vol. 82 10819281

[6] Park J-K, Seon J-K, Cho K-J, Lee N-H, Song E-K (2018) Is immediate postoperative mechanical Axis associated with the revision rate of primary total knee arthroplasty?? A 10-Year Follow-up study. Clin Orthop Surg 10:167–173. 10.4055/cios.2018.10.2.167 29854339 10.4055/cios.2018.10.2.167 PMC5964264

[7] Lotke PA, Ecker ML (1977) Influence of positioning of prosthesis in total knee replacement. J Bone Joint Surg Am 59:77–79 833180

[8] Benjamin J (2006) Component alignment in total knee arthroplasty. Instr Course Lect 55:405–412 16958475

[9] Bellemans J, Colyn W, Vandenneucker H, Victor J (2012) The Chitranjan Ranawat award: is neutral mechanical alignment normal for all patients? The concept of constitutional varus. Clin Orthop Relat Res 470:45–53. 10.1007/s11999-011-1936-5 21656315 10.1007/s11999-011-1936-5 PMC3237976

[10] Berend ME, Ritter MA, Meding JB, Faris PM, Keating EM, Redelman R et al (2004) Tibial component failure mechanisms in total knee arthroplasty. Clin Orthop Relat Res 26–34. 10.1097/01.blo.0000148578.22729.0e 10.1097/01.blo.0000148578.22729.0e 15534515

[11] Sadoghi P, Vendittoli P-A, Lustig S, Leal J, Graichen H, Rivière C et al (2022) Less religion and more science in the discussion of personalized alignment in total knee arthroplasty: we need to lead the transition process! Knee Surg Sports Traumatol Arthrosc 30:2883–2885. 10.1007/s00167-022-07079-z 35906411 10.1007/s00167-022-07079-z

[12] Milligan DJ, O’Brien S, Doran E, Gallagher NE, Beverland DE (2019) Twenty-year survivorship of a cemented mobile bearing total knee arthroplasty. Knee 26:933–940. 10.1016/j.knee.2019.06.004 31262634 10.1016/j.knee.2019.06.004

[13] van Ooij B, de Keijzer DR, Hoornenborg D, Sierevelt IN, Haverkamp D (2022) Lower revision rates for cemented fixation in a long-term survival analysis of three different LCS designs. Knee Surg Sports Traumatol Arthrosc 30:2707–2713. 10.1007/s00167-021-06587-8 33934194 10.1007/s00167-021-06587-8

[14] Couraudon A, Capdevielle P, Gedor M, Roche O, Sirveaux F, Mainard D (2025) Return to work after primary total knee replacement in patients under 55 years of age: a retrospective study of 129 cases. Orthop Traumatol Surg Res 104161. 10.1016/j.otsr.2025.104161 10.1016/j.otsr.2025.104161 39805549

[15] Kastner N, Gruber G, Aigner BA, Friesenbichler J, Pechmann M, Fürst F et al (2012) Sex-related outcome differences after implantation of low-contact-stress mobile-bearing total knee arthroplasty. Int Orthop 36:1393–1397. 10.1007/s00264-012-1486-9 22270864 10.1007/s00264-012-1486-9 PMC3385906

[16] Kastner N, Sternbauer S, Friesenbichler J, Vielgut I, Wolf M, Glehr M et al (2014) Impact of the tibial slope on range of motion after low-contact-stress, mobile-bearing, total knee arthroplasty. Int Orthop 38:291–295. 10.1007/s00264-013-2242-5 24346515 10.1007/s00264-013-2242-5 PMC3923942

[17] Ewald FC (1989) The knee society total knee arthroplasty roentgenographic evaluation and scoring system. Clin Orthop Relat Res. 248:9–2. 2805502

[18] Napier RJ, O’Neill C, O’Brien S, Doran E, Mockford B, Boldt J et al (2018) A prospective evaluation of a largely cementless total knee arthroplasty cohort without patellar resurfacing: 10-year outcomes and survivorship. BMC Musculoskelet Disord 19:205. 10.1186/s12891-018-2128-1 29945574 10.1186/s12891-018-2128-1 PMC6020353

[19] O’Brien S, Spence DJ, Ogonda LO, Beverland DE (2012) LCS mobile bearing total knee arthroplasty without patellar resurfacing. Does the unresurfaced patella affect outcome? Survivorship at a minimum 10-year follow-up. Knee 19:335–338. 10.1016/j.knee.2011.07.002 21856160 10.1016/j.knee.2011.07.002

[20] Buechel FF (2002) Long-term followup after mobile-bearing total knee replacement. Clin Orthop Relat Res 40–50. 10.1097/00003086-200211000-00008 10.1097/00003086-200211000-00008 12439236

[21] Solarino G, Spinarelli A, Carrozzo M, Piazzolla A, Vicenti G, Moretti B (2014) Long-term outcome of low contact stress total knee arthroplasty with different mobile bearing designs. Joints 2:109–114. 10.11138/jts/2014.2.3.109 25606553 10.11138/jts/2014.2.3.109 PMC4295681

[22] Lauretani F, Russo CR, Bandinelli S, Bartali B, Cavazzini C, Di Iorio A et al (2003) Age-associated changes in skeletal muscles and their effect on mobility: an operational diagnosis of sarcopenia. J Appl Physiol 95:1851–1860. 10.1152/japplphysiol.00246.2003 14555665 10.1152/japplphysiol.00246.2003

[23] Deeg DJLPHTBL (2000) Skeletal muscle mass and muscle strength in relation to lower-extremity performance in older men and women. Aging Male 3:161–161. 10.1080/13685530008500331 10.1111/j.1532-5415.2000.tb04694.x 10798463

[24] Sallinen J, Stenholm S, Rantanen T, Heliövaara M, Sainio P, Koskinen S (2010) Hand-Grip strength cut points to screen older persons at risk for mobility limitation. J Am Geriatr Soc 58:1721–1726. 10.1111/j.1532-5415.2010.03035.x 20863331 10.1111/j.1532-5415.2010.03035.x PMC2946262

[25] Mizner RL, Stevens JE, Snyder-Mackler L (2002) Preoperative quadriceps strength predicts functional outcome after total knee arthroplasty J Geriatr Phys Ther 25:42. 10.1519/00139143-200225030-00047

[26] Rantanen T (1999) Midlife hand grip strength as a predictor of old age disability. JAMA 281:558. 10.1001/jama.281.6.558 10022113 10.1001/jama.281.6.558

[27] Hashimoto S, Hatayama K, Terauchi M, Saito K, Higuchi H, Chikuda H (2020) Preoperative hand-grip strength can be a predictor of stair ascent and descent ability after total knee arthroplasty in female patients. J Orthop Sci 25:167–172. 10.1016/j.jos.2019.03.003 30904204 10.1016/j.jos.2019.03.003

[28] Kakiage H, Hatayama K, Nonaka S, Terauchi M, Saito K, Takase R et al (2025) Stair climbing ability and postoperative activity in patient-reported outcomes after CR-TKA are more related to handgrip strength than sagittal knee stability. Arch Orthop Trauma Surg 145:113. 10.1007/s00402-024-05678-8 39776240 10.1007/s00402-024-05678-8

